# Rejecting unfairness: emotion-driven reaction or cognitive heuristic?

**DOI:** 10.3389/fnhum.2013.00126

**Published:** 2013-04-05

**Authors:** Claudia Civai

**Affiliations:** Department of Economics, College of Liberal Arts, University of MinnesotaMinneapolis, MN, USA

In the following paragraphs, I am arguing that rejecting inequality, even when it means sacrificing available resources, could be interpreted as a default response that occurs when there is no other reason to choose otherwise. Moreover, I am reviewing some of our latest findings suggesting that emotions might not be the sole mechanism that ultimately explains this response, as claimed instead by the most accredited account (e.g., Sanfey et al., 2003; van't Wout et al., 2006; Crockett et al., 2008; Tabibnia et al., 2008). The idea that a 50-50 share is preferred over other distributions, when there is no reason to support one of the contending parties, is not new to the psychological debate: it has been suggested that people use equality heuristically, because it has psychological advantages, such as being a cognitive simple strategy, easy to use and to be understood by everyone, quickly implemented, defensible, and, moreover, a useful starting point from which, in case, adjustments can be made (Messick and Schell, 1992; Messick, 1995). Furthermore, less-equal distributions are consistently rejected more often among different human populations (Henrich et al., 2006). The central claim of Bicchieri's book *The Grammar of Society* (2006) is that an equal-division norm plays a critical and under-appreciated role in driving behavior in bargaining games (Bicchieri, 2006; Nichols, 2010). Research in the field of behavioral economics has demonstrated that the model of *homo economicus* often fails to predict human behavior: the Ultimatum Game (UG) (Güth et al., 1982), a widely employed tool to investigate socio-economic decision-making, perfectly shows how people do not always make decisions driven by the principle of maximizing monetary payoff. In this game, a proposer has to share some money with a responder, who can either accept or reject the offer: if he accepts, the money is divided as the proposer has established, otherwise both of them get nothing. To maximize their payoffs, the proposer should offer the smallest amount of money, and responder should always accept, as even one is better than zero. However, numerous findings show that the proposer tends to make fair offers, around 50% of the share, while the responder prefers to reject a sure amount of money rather than accepting an unfair division. Models of social preferences provide a formal explanation for the apparently irrational behavior. Two families of theories, i.e., theories of negative reciprocity (e.g., Rabin, 1993; Falk and Fischbacher, 2006) and theories of inequality aversion (e.g., Fehr and Schmidt, 1999) tried to explain rejections: the former focuses on intentions and describes rejections as a tool to punish the unfair proposer, whereas the latter focuses on the outcome and claims that people are naturally averse to unequal distributions, especially when disadvantageous. Recently, Tricomi et al. (2010) found support for this claim, showing that basic reward brain structures, such as ventral striatum and ventromedial prefrontal cortex (vMPFC), are involved in both advantageous and disadvantageous inequity. From a psychological perspective, negative emotions, such as anger and frustration, elicited by the unfair treatment, are accounted to cause rejections (Pillutla and Murnighan, 1996), and a number of neuroscientific findings support this hypothesis: for example, van't Wout et al. (2006), using the skin conductance response (SCR) as a measure of emotional activation, reported that people were more emotionally aroused, showing a higher SCR when rejecting, as opposed to accepting, unfair offers. Moreover, areas known to be involved in emotional control, such as vMPFC, and in processing negative emotions, such as anterior insula (AI), are found also to be activated by rejections, and not acceptances, of UG unfair offers (e.g., Sanfey et al., 2003; Koenigs and Tranel, 2007).

However, if it is true that the accounts described above, i.e., negative reciprocity, inequality aversion and emotional involvement, explain responder's behavior in the standard UG paradigm, it is hard to develop a psychological interpretation of broad inequality perception based on the evidence collected using this standard version. First of all, UG is a self-centered task: perception of unfairness is confounded with self-serving bias, questioning whether responder is actually rejecting disadvantageous outcomes rather than a general idea of unequal division; also, it is unclear if anger and frustration are elicited by unfairness or by self-involvement. Second, the proposer is always the source of the unfair division confounding outcome and intentions concerns. Many studies have addressed the issue of intentions (e.g., Sutter, 2007; Falk et al., 2008); in particular, Blount (1995) compares the rejection rate of allocations decided by either a person or an algorithm that shares a sum of money randomly between two players, finding higher rejection rates in the first case compared to the second. However, the demands of this task were different, in that the experimenter asked the participants to indicate their expectations on the two distributions prior to the choice period, and this may have biased the responses (Sanfey, 2009). Nonetheless, rejection rate for the algorithm condition was not zero, confirming that outcome still plays a role as well. Third, the proposer, who decides how to allocate the money, always benefits from one part of the share, thus the responder never faces outcomes which exceed the 50% of the pie, confounding rejections of unequal outcomes with rejections of disadvantageous payoffs, and leaving questions concerning advantageous inequality unanswered.

Our research aimed at understanding the nature of a general inequality aversion, if any, employing manipulations of the traditional UG. First, we addressed the issue of the self-serving bias, by asking participants to play as responders both for themselves (myself-MS-condition), and on behalf of a third-party (TP condition), in which their payoff is not affected by their decision. Borrowing a famous expression coined by Adam Smith in his work *The Theory of Moral Sentiments* (1759), this manipulation put the participant in the condition of the “impartial spectator,”^1^ in that the decision made by the participant affected someone else's pockets; this way, it was possible to disentangle between the two hypotheses, i.e., rejections and negative emotions as elicited by the perception of unfairness itself, or rejections and negative emotions as related to the fact of being the target of the unfair division. We employed this paradigm in two studies: in the first study, we recorded the behavior, as the percentage of rejected offers (RR), and the SCR, to get a measure of emotional arousal (Civai et al., 2010), and in the second study we investigated neural activation by the mean of functional magnetic resonance (fMRI) (Corradi-Dell'Acqua et al., 2012). In both studies, behavioral analysis showed no difference between MS and TP: specifically, RR was higher for unfair offers, and decreased as the offers became fairer, both in MS and in TP. However, behavior dissociated from both psychophysiological and neural activations. In the first study, participants were more aroused, showing higher SCR and higher subjective emotional ratings, when rejecting, compared to accepting, offers in MS, but not in TP, where, instead, there was no effect of response on SCR. These results suggested that, albeit emotional arousal clearly enters the decision-making process, it should not be held as being the only mechanism that triggers rejections, in that rejections occurred also when there was no sign of it. Neuroimaging data of the second study revealed a dissociation between the medial prefrontal cortex, specifically associated with rejections in MS condition, thus confirming its role in self-related emotional responses, and the left AI, associated with rejections in both MS and TP conditions, supporting the hypothesis of a role played by this area in promoting fair behavior also toward third-parties (Spitzer et al., 2007; King-Casas et al., 2008). In both studies, findings in TP condition support the idea that people are concerned about unfairness among others, as showed by previous studies (Fehr and Fischbacher, 2004).

In two subsequent studies, we asked responders to decide whether to accept or reject allocations made by an external proposer, which could be either a person or a random number generator; MS-TP manipulation was maintained. This design rules out the possibility of using rejections to punish the source of unfairness; the idea is that if rejections still occur, then they have to be driven by the outcome and not by the unfair intentions. Moreover, responders were presented with allocations which were unequal but, at the same time, advantageous for them, allowing disentangling between decisions on disadvantageous and advantageous inequality. In both studies, participants rejected unequal offers, showing to care about the outcome rather than specifically about the intentions. In particular, unequal allocations in TP were mostly rejected, as well as unequal disadvantageous offers in MS, but unequal advantageous offers were mostly accepted (Civai et al., 2012). Imaging results showed a higher activation of the MPFC for disadvantageous, as opposed to advantageous, offers in MS, but not in TP, and this activation was negatively correlated with rejections; activation in the AI, instead, was higher for unequal offers, both disadvantageous and advantageous, irrespectively of the target (MS and TP) (Civai et al., 2012). Behavioral results confirmed that people prefer equal divisions and care about equality among third-parties; however, these findings also suggest that people change their preference when involved in first person, accepting inequality when it brings them an advantage on the other player. In terms of neural activations, the involvement of MPFC in MS rejections was confirmed; this activation extended more dorsally with respect to the MPFC activation found in the previous imaging study, supporting a recent account which claims that dorsal MPFC may be involved in shifting preference from a default option, represented in this case by rejecting the outcome, to a new one (Boorman et al., 2013). Interestingly, AI was activated by the perception of inequality, and was by no means related to the advantageousness of the offer in MS, supporting the idea of a crucial role played this area in signaling deviations from the norm, or expected outcome (King-Casas et al., 2008; Xiang et al., 2013).

In conclusion, our findings support an account that considers the rejection of inequality as a cognitive heuristic, a psychological anchor, which is a useful starting point that can be easily adjusted when salient contextual cues enter the environment and influence the decision. In our studies, third-party condition can be considered as the neutral situation, designing a context in which participants have no particular reason to accept inequality, except for maximizing the total payoff; in this neutral condition, people apply the simple strategy of equal split. First-person involvement (MS condition) is a salient contextual cue that modifies the environment and shifts the preference from 50-50 shares to outcomes that favor the responder. This interpretation is in line with recent findings about expectations, which showed that participants are more prone to reject offers when primed with expectations of fairness (Sanfey, 2009); moreover, a formal model that considers expectations outperforms models of inequity aversion in predicting behavior (Chang and Sanfey, 2013). In this framework, expectations can be considered as the contextual cues that shift preferences away from the default 50-50. Interestingly, it seems that emotional arousal is limited to disadvantageous unfairness; however, the rejection of equality norm's violation occurs despite the lack of emotional arousal, suggesting the cognitive nature of the equal split heuristic. As far as the neural correlates are concerned, results suggest that the activation of AI in the UG be interpreted as a signal of deviation from an expected outcome (Chang et al., 2013; Xiang et al., 2013), which is, in this case, the equal split, rather than just a sign of emotional arousal; this interpretation also offers a straightforward and parsimonious way to account for the variety of cognitive and emotional tasks in which the AI has been found to play a role (see Craig, 2009 for a review on the tasks).

## Footnotes

^1^Although the semantic expression in this context is appropriate, the concept of impartial spectator as described by Adam Smith in his work The Theory of Moral Sentiments is different: here, the author argues that, in order to go beyond our own presuppositions to judge a situation, we do not have to rely literally on a third-party impartial spectator, but rather we have to “remove ourselves, as it were, from our own natural situation, and endeavor to view them at a certain distance from us” (Smith, The Theory of Moral Sentiments, 1759, III, I, 2).
